# Upregulation of arylsulfatase B in carotid atherosclerosis is associated with symptoms of cerebral embolization

**DOI:** 10.1038/s41598-017-04497-9

**Published:** 2017-06-28

**Authors:** Erik Biros, Corey S. Moran, Jane Maguire, Elizabeth Holliday, Christopher Levi, Jonathan Golledge

**Affiliations:** 1 The Queensland Research Centre for Peripheral Vascular Disease, College of Medicine and Dentistry, James Cook University, Townsville, Queensland Australia; 2 School of Nursing and Midwifery, University of Newcastle, Callaghan, NSW Australia; 3 Public Health Research Program, Hunter Medical Research Institute, Newcastle, NSW Australia; 4 John Hunter Hospital, Hunter Medical Research Institute and University of Newcastle, Callaghan, NSW Australia; 5 Department of Vascular and Endovascular Surgery, The Townsville Hospital, Townsville, Queensland Australia

## Abstract

The aim of this study was to identify genes for which the expression within carotid atherosclerosis was reproducibly associated with the symptoms of cerebral embolization. Two publically available microarray datasets E-MEXP-2257 and GSE21545 were analysed using GeneSpring 11.5. The two datasets utilized a total of 22 and 126 carotid atherosclerosis samples, obtained from patients with and without symptoms of cerebral embolization, respectively. To assess whether the findings were reproducible we analysed carotid atherosclerosis samples from another 8 patients with and 7 patients without symptoms of cerebral embolization using real-time PCR. *In vitro* studies using VSMC were performed to assess the functional relevance of one of the validated genes. We identified 1624 and 135 differentially expressed genes within carotid atherosclerosis samples of symptomatic compared to asymptomatic patients using the E-MEXP-2257 and GSE21545 datasets, respectively (≥1.15-absolute fold-change, P < 0.05). Only 7 differentially expressed genes or 0.4% (7/1,752) were consistent between the datasets. We validated the differential expression of *ARSB* which was upregulated 1.15-fold (P = 0.029) in atherosclerosis from symptomatic patients. *In vitro* incubation of VSMCs with the ARSB inhibitor L-ascorbic acid resulted in marked upregulation of *SIRT1* and *AMPK*. This study suggests that ARSB may represent a novel target to limit carotid embolization.

## Introduction

The prevalence of carotid artery stenosis is approximately 4% to 8% in adults aged 50 to 79 years^1–3^. Carotid atherosclerosis is estimated to be responsible for ~20% of all ischemic strokes^4, 5^. Atherosclerotic plaque rupture and cerebral embolization is believed to be the mechanism by which carotid atherosclerosis leads to cerebral symptoms, such as transient ischemic attack and stroke^1^. However, the identification of the so-called “vulnerable plaque” has been elusive. The first whole-genome gene expression study of stroke was published a decade ago^6^. The authors at that time profiled peripheral blood mononuclear cells of stroke patients and compared them with those of healthy donors^6^. Almost 200 differentially expressed genes were identified; however, those assessed had limited diagnostic value with estimated specificity and sensitivity less than 80%^6^. A number of previous studies have examined differential gene expression in symptomatic carotid atherosclerosis; however, findings have not been consistent across the studies^7–15^. All these results question the value of differential gene expression in representing true molecular determinants of stroke. The current study re-examined previously published microarray datasets of carotid artery atherosclerosis to gain further insight. An attempt has been made to identify consistent and reproducible differentially expressed genes using publically available microarray datasets that utilized carotid endarterectomy samples from symptomatic and asymptomatic patients.

## Methods

### Data preparation

Two human microarray datasets were included in this study to establish a consensus set of differentially expressed genes in carotid atherosclerosis associated with the recent symptoms of cerebral embolization^12, 13^. Suitable datasets were required to be publically available, to assess whole-genome gene expression within carotid plaque biopsies from patients both with and without symptoms of cerebral embolization, and to use commercially available microarrays of the same chip platform. The publically available raw array data were downloaded from Gene Expression Omnibus (GEO) provided by the National Center for Biotechnology Information (NCBI) or ArrayExpress provided by the European Molecular Biology Laboratory-European Bioinformatics Institute (EMBL-EBI) public repositories.

### Re-analysis of the original array data

In order to identify differentially expressed genes in carotid atherosclerotic tissue between symptomatic and asymptomatic patients, we analysed each original microarray dataset as described previously^16^. Briefly, the raw data matrix downloaded from GEO or ArrayExpress public repositories was uploaded into GeneSpring GX 11.5 (Agilent Technologies Pty Ltd) and the standard normalization procedures recommended for the Affymetrix GeneChip arrays was followed. Expression values were normalized using percentile shift normalization with default settings. These included normalization to 75^th^ percentile. The expression profile of carotid atherosclerosis samples obtained from participants with symptoms of cerebral embolization was compared to those without symptoms. Since all samples represented advanced atherosclerotic tissue, only small differences in gene expression were expected between patients with and without symptoms of cerebral embolization. Previous evidence suggested that small changes in gene expression are able to induce significant phenotypic differences^17^. In line with this, genes with ≥1.15-absolute fold differential expression between groups based on P-value < 0.05 by non-parametric Mann–Whitney U-test with no mathematical correction for multiple testing were considered to be differentially expressed.

### Validation of microarrays findings

Using total RNA obtained from atherosclerosis removed from the proximal internal carotid (PIC) arteries of 8 patients with and 7 patients without symptoms of cerebral embolization, we assessed the validity of microarray findings (validation group). Total RNA was extracted from PIC biopsies stored in RNAlater (Sigma-Aldrich) at −80 °C using RNeasy Mini Kit (Qiagen) according to manufacturer’s instructions. Symptomatic patients presented with focal neurological symptoms related to their anterior cerebral circulation such as transient ischemic attack (TIA) or stroke within 6 weeks of surgery; asymptomatic patients presented with no history of neurological symptoms^1^. Quantitative real-time reverse transcription PCR (qRT-PCR) assay was performed to assess the relative expression of arylsulphatase B (*ARSB*) as this was consistently differential expressed in the array analyses. The relative expression of *ARSB* in each sample was calculated by using the Concentration-Ct-standard curve method and normalized using the average expression of the glyceraldehyde-3-phosphate dehydrogenase (*GAPDH*) gene for each sample using the Rotor-Gene Q operating software version 2.0.24 (Qiagen). *GAPDH* was chosen as the “housekeeping” gene since analyses showed its expression to be similar in carotid biopsies from symptomatic and asymptomatic patients. The QuantiTect SYBR Green one-step RT-PCR Kit (Qiagen) was used according to the manufacturer’s instructions with 20 ng of total RNA as template. All reactions were independently repeated in duplicate to assess the repeatability of the results and the mean of the two values for each sample was used for analyses. The QuantiTect Primer Assays QT00026684 and QT00079247 (Qiagen) were used for the *ARSB* and *GAPDH* assessments, respectively. Mann–Whitney U test was performed to identify differences in expression levels of *ARSB* between carotid atherosclerosis biopsies of patients with and without symptoms of cerebral embolization. Data were presented as box-and-whisker plots with median and interquartile range with maximum and minimum data points (whiskers) for relative expression. Statistical significance was defined at the conventional 5% level. All computations were performed using the Stata/MP 13.1 statistical software (StataCorp LP, USA). Ethical approval was granted from The Townsville Hospital (TTH) and Health Services Committees, written informed consent was obtained from each participant, and the protocols conformed to the ethical guidelines of the Declaration of Helsinki.

### Cell culture

We investigated the effects of chondroitin sulphate, a natural substrate for ARSB, and L-ascorbic acid, an ARSB inhibitor, on the expression of sirtuin 1 (*SIRT1*) and protein kinase AMP-activated catalytic subunit alpha 1 (*PRKAA1* or *AMPK*, 5′-prime-AMP-activated protein kinase) under inflammatory conditions *in vitro*. These two ARSB-associated bioactive molecules were specifically selected to test their individual and combined ability to upregulate the SIRT1/AMPK metabolic pathway that exerts strong anti-inflammatory, anti-atherogenic, and plaque-stabilizing effects^18, 19^. We used human vascular smooth muscle cells (VSMC; Clonetics) that were plated at a seeding density of 1 × 10^5^ cells/well and maintained at 37 °C, 5% CO_2_, in Dulbecco’s Modified Eagle Medium (DMEM; Sigma-Aldrich) containing 10% fetal bovine serum (FBS; Gibco). Cells were growth arrested at 90% confluency by incubation in DMEM + 0.1% FBS overnight (18 hours). Control cultures (n = 6) were exposed to DMEM + 10% FBS comprising 10% v/v conditioned media generated from human monocytic THP-1 cells exposed to 10 µg/ml endotoxin (Lipopolysaccharide; Sigma-Aldrich) over a period of 24 hours. Experimental cultures were exposed to the same pro-inflammatory media but supplemented with either L-ascorbic acid (Sigma-Aldrich; 400 μM; n = 6 cultures) or chondroitin sulphate sodium salt (Sigma-Aldrich; 300 μM; n = 6 cultures), or a combination of both (n = 6 cultures). All cells were harvested after a 24-hour experimental period and subjected to RNA extraction followed by qRT-PCR using the Qiagen’s QuantiTect Primer Assay QT00009436 (*AMPK*) and QT00051261 (*SIRT1*) as outlined above.

## Results

### Datasets characteristics

Two whole-genome gene expression datasets were included in this study to determine the consensus set of differentially expressed genes in carotid atherosclerosis associated with the symptoms of cerebral embolization (Table 1). The E-MEXP-2257 dataset utilized 22 carotid plaque biopsies obtained from 13 and 9 patients with and without symptoms of cerebral embolization, respectively. The summary characteristics of participants included in the E-MEXP-2257 dataset are presented elsewhere^12^. In brief, the mean age of patients was 64 ± 8 years, ~27% of participants were females (6/22), and ~27% of participants (6/22) had a positive history of smoking (Table 1). The second dataset included in this study, GSE21545, utilized 126 carotid plaque biopsies obtained from 25 and 101 symptomatic and asymptomatic patients, respectively (Table 1). The summary characteristics of participants included in the GSE21545 dataset are presented elsewhere^13^. Briefly, the mean age of patients was 71 ± 9 years, ~22% of participants were females (28/126), and ~49% of participants (62/126) had a positive history of smoking (Table 1). No individual patient’s clinical characteristics, including medication and severity of carotid atherosclerosis, were publically available for both datasets.

**Table 1:** Characteristics of two microarray datasets included in this study.

Data set	E-MEXP-2257	GSE21545
Reference	12	13
Technology used	Affymetrix GeneChip HG-U133A	Affymetrix GeneChip HG-U133 Plus 2
Number of transcripts analysed	18,400	47,000
Sample analysed	Carotid plaque biopsies	Carotid plaque biopsies
Number of patients	22	126
Number of symptomatic patients	13 (59%)	25 (20%)
Age (years)	64 ± 8	71 ± 9
Number of females	6 (27%)	28 (22%)
Number of current or previous smokers	6 (27%)	62 (49%)

Age, calendar age at entry-to-study presented as mean ± standard deviation (SD).

### Numerical assessment of differentially expressed genes

**Figure 1:**
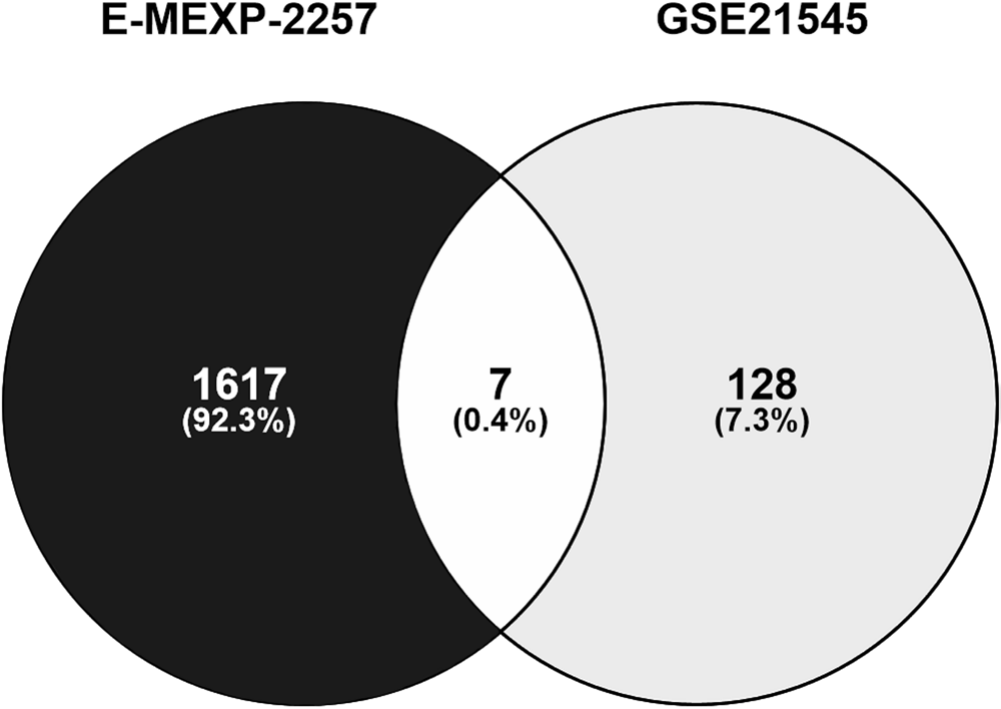
Numerical assessment of genes for which the expression in carotid atherosclerosis was associated with the symptoms of cerebral embolization. Venn diagram illustrating the overlap between the E-MEXP-2257 and GSE21545 microarray datasets profiling differentially expressed genes within carotid plaque biopsies. Samples obtained from patients with symptoms of cerebral embolization were compared with those of patients without the symptoms (≥1.15-absolute fold difference, P < 0.05 without mathematical correction for multiple comparisons calculated with non-parametric Mann-Whitney test).

The E-MEXP-2257 and GSE21545 microarray datasets were individually re-analysed to identify differentially expressed genes and the overlap between the findings. A total of 1,624 and 135 genes were found to be differentially expressed (≥1.15-absolute fold change, uncorrected P < 0.05) within carotid plaques of symptomatic compared to asymptomatic patients in E-MEXP-2257 and GSE21545, respectively (Fig. 1). Full lists of differentially expressed genes are given in Supplemental Table I and Supplemental Table II for E-MEXP-2257 and GSE21545, respectively. Although E-MEXP-2257 and GSE21545 collectively identified 1,752 differentially expressed individual genes, only 7 genes or 0.4% were consistently differentially expressed in the two datasets (7/1,752; Fig. 1).

### Assessment of consistently differentially expressed genes

Inspection of the differentially expressed genes revealed that the arylsulfatase B (*ARSB*) gene was the only gene consistently upregulated in symptomatic compared to asymptomatic patients identified in both E-MEXP-2257 (1.15-fold change, P = 0.039) and GSE21545 (1.15-fold change, P = 0.030) datasets (Table 2). The coagulation factor III, tissue factor (*F3*) gene and the ubiquitin like modifier activating enzyme 6 (*UBA6*) gene were found to be upregulated in symptomatic compared to asymptomatic patients in the E-MEXP-2257 dataset but downregulated in symptomatic compared to asymptomatic patients in the GSE21545 dataset (Table 2). The growth arrest specific 6 (*GAS6*) gene, the uncharacterized LOC730101 (*LOC730101*), and the secretory leukocyte peptidase inhibitor (*SLPI*) gene were identified to be downregulated in symptomatic compared to asymptomatic patients in both datasets (Table 2). Finally, the G protein-coupled receptor 135 (*GPR135*) gene was found to be downregulated in symptomatic compared to asymptomatic patients in the E-MEXP-2257 dataset but upregulated in symptomatic compared to asymptomatic patients in the GSE21545 dataset (Table 2).

**Table 2:** Genes for which the expression in carotid atherosclerosis was consistently associated with the symptoms of cerebral embolization in the two microarray datasets included in this study.

Gene symbol	Gene name	E-MEXP-2257 dataset			GSE21545 dataset		
		Fold change	Regulation	P-value	Fold change	Regulation	P-value
*ARSB*	arylsulfatase B	1.15	Up	0.039	1.15	Up	0.030
*F3*	coagulation factor III, tissue factor	1.26	Up	0.019	−1.20	Down	0.035
*GAS6*	growth arrest specific 6	−1.42	Down	0.011	−1.18	Down	0.032
*GPR135*	G protein-coupled receptor 135	−1.21	Down	0.004	1.17	Up	0.012
*LOC730101*	uncharacterized LOC730101	−1.16	Down	0.006	−1.20	Down	0.025
*SLPI*	secretory leukocyte peptidase inhibitor	−1.27	Down	0.013	−1.35	Down	0.018
*UBA6*	ubiquitin like modifier activating enzyme 6	1.20	Up	0.039	−1.39	Down	0.032

P-value, calculated with non-parametric Mann-Whitney test without mathematical correction for multiple comparisons.

### Validation of microarray findings

**Figure 2:**
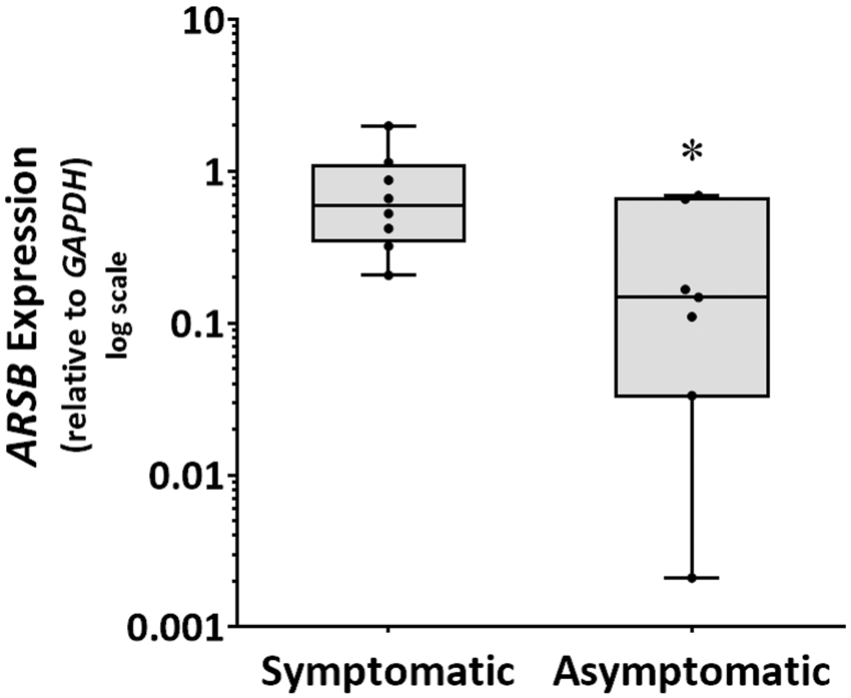
Expression of the *ARSB* gene within carotid atherosclerosis of patients with and without symptoms of cerebral embolization. Increased expression of *ARSB* within carotid atheroma biopsies of symptomatic (N = 8) compared to asymptomatic (N = 7) patients (*P = 0.029). Data expressed as median and interquartile range with maximum and minimum data points (whiskers) for relative expression and compared by Mann-Whitney U test.

The validity of microarray findings was further assessed using carotid atherosclerosis biopsies obtained from 8 symptomatic and 7 asymptomatic patients undergoing carotid endarterectomy (validation group; Table 3). The risk factors and medications of symptomatic and asymptomatic patients were similar (Table 3). The relative expression of *ARSB*, the only gene consistently upregulated in symptomatic compared to asymptomatic patients in both microarray datasets, was also found to be significantly increased within the carotid atherosclerotic tissue of symptomatic compared to asymptomatic patients of the validation group using qRT-PCR (*P = 0.029; Fig. 2).

**Table 3:** Risk factors and medication of patients with and without symptoms of cerebral embolization included in the validation group.

Characteristic	Symptomatic patients	Asymptomatic patients	P-value
Number of patients	8	7	—
Age (years)	72 ± 11	72 ± 5	0.779
Number of females	2 (25%)	1 (14%)	0.677
Number of current or previous smokers	7 (88%)	6 (86%)	0.933
Type 2 diabetes	3 (38%)	1 (14%)	0.390
Hypertension	6 (75%)	5 (71%)	0.962
Ischemic heart disease	3 (38%)	3 (43%)	0.853
Dyslipidaemia	5 (63%)	4 (57%)	0.853
Statins	5 (63%)	3 (43%)	0.505
Fibrates	0 (0%)	2 (29%)	0.200
Angiotensin converting enzyme inhibitors	2 (25%)	5 (71%)	0.109
Angiotensin receptor blockers	2 (25%)	1 (14%)	0.462

Nominal variables are presented as numbers; continuous variables are presented as mean ± standard deviation. Two-sided P value calculated by Mann Whitney U test (continuous variables) or Fisher’s exact test (nominal variables).

### Cell culture

**Figure 3:**
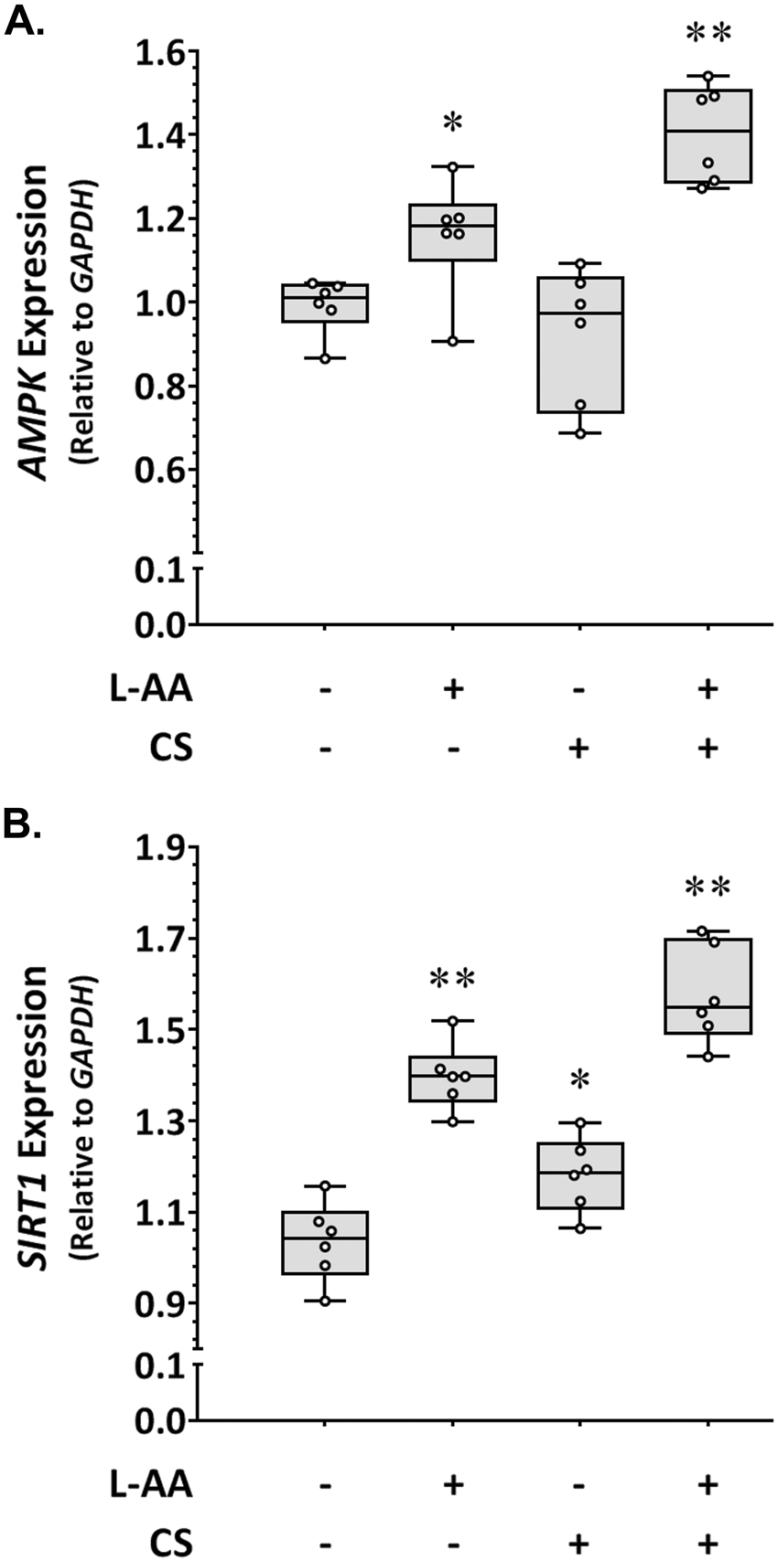
Effect of L-ascorbic acid and chondroitin sulphate on *AMPK* and *SIRT1* expression in inflammation-activated VSMC *in vitro*. Upregulation of *AMPK* (**A**) (*P = 0.041, **P = 0.002) and *SIRT1* (**B**) (*P = 0.015, **P = 0.002) in VSMCs incubated with L-ascorbic acid (L-AA) and chondroitin sulphate (CS) compared to control. Data expressed as median and interquartile range with maximum and minimum data points (whiskers) for relative expression and compared by Mann-Whitney U test.

Finally, we investigated the effect of chondroitin sulphate (ARSB substrate) and L-ascorbic acid (ARSB inhibitor) on important anti-atherogenic pathways represented by the *AMPK* and *SIRT1* genes using human VSMC *in vitro* under inflammatory conditions. We found that incubation of VSMC with L-ascorbic acid was associated with upregulation of both *AMPK* (Fig. 3A) and *SIRT1* (Fig. 3B). The incubation of VSMC with chondroitin sulphate was associated with upregulation of *SIRT1* (Fig. 3B) but not *AMPK* (Fig. 3A). Importantly, the simultaneous incubation of VSMC with chondroitin sulphate and L-ascorbic acid resulted in synergistic upregulation of *AMPK* (Fig. 3A) and additive upregulation of *SIRT1* (Fig. 3B). These findings suggest that the combination of chondroitin sulphate and L-ascorbic acid may represent a potent activator of the AMPK/SIRT1 pathways.

## Discussion

We analysed publically available microarray datasets from previous gene expression studies that utilised carotid plaque biopsies from patients with and without symptoms of cerebral embolization^12, 13^. By focusing on genes that were simultaneously differentially expressed in the datasets analysed, we were able to discover and validate the upregulation of *ARSB* in carotid plaques of symptomatic compared to asymptomatic patients, not acknowledged in the original microarray studies. It is important to note that the protein product of this gene is involved in degradative processes of sulphated proteoglycans, the major component of virtually all extracellular matrices (ECMs)^20^. Furthermore, previous data suggest an inverse association between enzymatic activity of ARSB and the stability of sulphated proteoglycans within the ECM^21^. In line with this, Koledgie *et al*. found that plaque rupture sites contain very little proteoglycan content relative to stable lesions^22^, consistent with a degradative process.

Although the upregulation of *ARSB* within the carotid plaque biopsies of symptomatic patients identified in both microarray datasets was relatively small, we were able to confirm these findings by qRT-PCR using carotid plaque biopsies obtained from another group of symptomatic and asymptomatic patients. This led us to hypothesize that upregulation of *ARSB* may represent an important pathological mechanism associated with symptoms of cerebral embolization, consistent with previous findings that even small changes in gene expression can induce major phenotypic differences^17^. The ARSB enzyme catalyses de-sulphation of ubiquitous glycosaminoglycans such as chondroitin sulphate^23^. Published evidence suggests that plasma concentration of under-sulphated chondroitin is elevated in patients with carotid artery disease^24^, while sulphated chondroitin has been long known to exhibit anti-atherogenic properties in rodents, primates, and humans^25–28^. Several historical studies from the 1960s and 1970s report reduced incidence of coronary events and cardiovascular mortality in atherosclerotic subjects treated with chondroitin sulphate^28–30^. Recent data suggests that anti-atherogenic actions of chondroitin sulphate may occur through interfering with the pro-inflammatory activation of monocytes and endothelial cells by tumor necrosis factor (TNF) alpha^31^, a cytokine thought to be crucially involved in the pathogenesis of atherosclerotic plaque^32^. Although the authors did not elucidate the exact mechanism of action of chondroitin sulphate^31^, it is possible that upregulation of anti-inflammatory microRNAs, the negative regulators of gene expression, could play a role^33–35^. Previous studies suggest an inhibitory effect of chondroitin sulphates on gene expression through modification of microRNAs^36^. Importantly, the ARSB enzyme is inhibited by L-ascorbic acid^21, 37^. Due to the lack of a well-developed animal model of carotid atherosclerosis associated with cerebral embolization, we further investigated the effect of L-ascorbic acid and chondroitin sulphate on important anti-atherogenic pathways *in vitro*. We found that chondroitin sulphate and L-ascorbic acid administered together induced a remarkable upregulation in the expression of *SIRT1* and *AMPK* genes in VSMCs exposed to inflammatory conditions *in vitro*. These findings suggest that chondroitin sulphate formulated with L-ascorbic acid may serve as a potent activator of the SIRT1/AMPK pathway. This may hold promise as a novel therapeutic approach for carotid atherosclerosis since the SIRT1/AMPK pathway is key to a number of vasculoprotective processes. In particular, SIRT1 is the nicotinamide adenosine dinucleotide (NAD)-dependent deacetylase that has been associated with inhibition of the proatherogenic VSMC foam cell formation possibly through the suppressing of the nuclear factor kappa B (NF-κB) signalling pathway^38^. AMPK is the main energy-sensing kinase in all eukaryotic cells and has been implicated in stabilizing atherosclerotic plaques through the inhibition of the mammalian target of rapamycin (mTOR) signalling pathway^39^. The downregulation of genes for secretory leukocyte peptidase inhibitor (*SLPI*), uncharacterized *LOC730101*, and growth arrest specific 6 (*GAS6*) in carotid atherosclerosis associated with the symptoms of cerebral embolization in both datasets included in this study was also demonstrated. The role of *SLPI* and *LOC730101* in human carotid atherosclerosis is largely unknown. Some evidence suggests that more severe atherosclerosis in humans is associated with an increase in *GAS6* expression^40^, while similar expression of *GAS6* in human carotid arteries with and without atherosclerosis has been reported^41^. Further investigation of the role of these three genes in carotid atherosclerosis is needed.

The limitations of this study include the relatively small number of patients included in the original microarray datasets. In particular, although both datasets collectively included 148 carotid atherosclerosis tissue samples, the total number of 38 ischemic events was relatively small and findings need to be substantiated by larger studies. We also observed very limited overlap and consistency between the genes differentially expressed in the two datasets suggesting the heterogeneous nature of the patients investigated. Further genome-wide gene expression studies involving histologically standardized sets of patients are needed. In view of these limitations we sought to validate important microarrays finding using another set of carotid artery biopsies obtained from patients with and without symptoms of cerebral embolization. The assessment of independent samples helps to minimize the possibility that selection biases adversely affected the generalizability of the findings. We did, however, only assess mRNA not protein levels due to limited availability of carotid artery biopsies. Finally, the exact mechanism by which chondroitin sulphate combined with L-ascorbic acid upregulated the SIRT1/AMPK pathway as well as the therapeutic doses of these two bioactive molecules remains to be elucidated.

In conclusion, a decade after the first microarrays for stroke, its molecular determinants are still poorly understood. The outcome of this study highlight a potential role for arylsulfatase B in promoting atherosclerosis-related stroke and warrants its further investigation as a therapeutic target that could be of potential clinical benefit.

## Electronic supplementary material

Supplemental Table I

Supplemental Table II
